# Turning water into rock: The inverted waves effect

**DOI:** 10.1177/2041669515627951

**Published:** 2016-02-01

**Authors:** Jukka Häkkinen, Lauri Gröhn

**Affiliations:** Institute of Behavioural Sciences, University of Helsinki, Finland; Synestesia Software Music, Finland

**Keywords:** shape from shading, materials, surface perception, perception of water

## Abstract

Humans perceive shape in two-dimensional shaded images, and turning such an image upside down can result in inversion of the relief of this image. Previous research indicates that this inversion is caused by assumptions related to overhead illumination, global convexity and viewpoint above the surface. In our article, we describe the inverted waves effect, in which turning an image of waves upside down changes its relief and also its perceived material properties.

If there is a limited amount of information about scene structure, the visual system makes assumptions to reach an interpretation of a visual scene. For example, when circular patches containing a shading gradient, such as that in Figure 1(a), are viewed, the observer perceives the surrounding patches as convex bulges and the central patch as a concave indentation. If the image is turned upside down, as in Figure 1(b), the three-dimensional (3D) interpretation of the image changes: the concave areas become convex, and the convex areas become concave. This is a well-known effect (Brewster, 1826; Rittenhouse, 1786; von Fieandt, 1949) that is related to the visual system’s assumption that the light source is above the image (Ramachandran, 1988).

**Figure 1.**
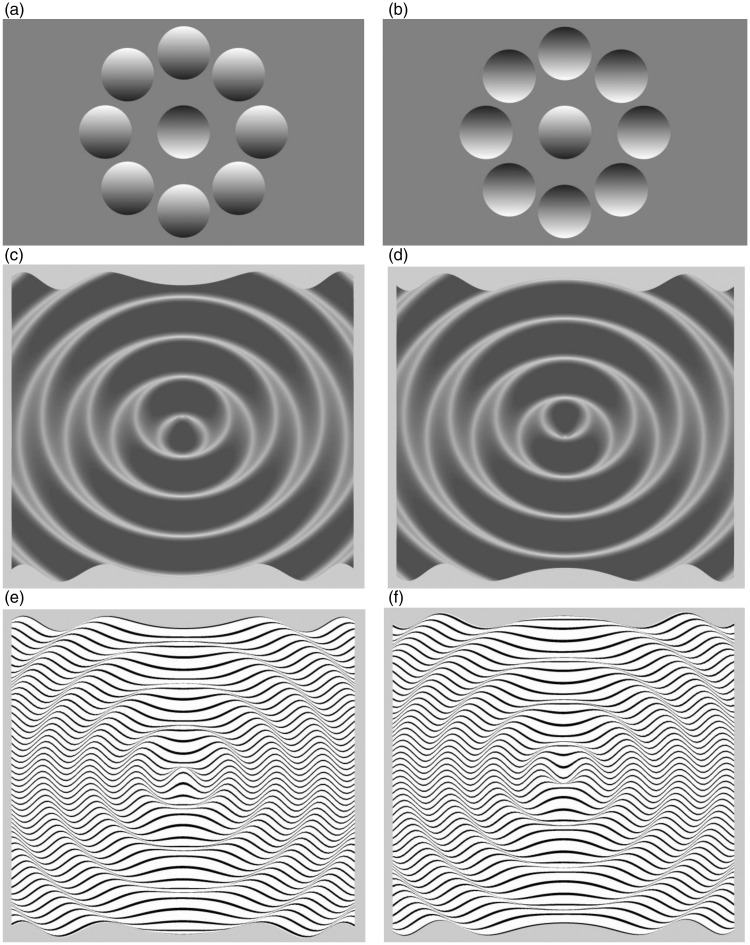
(a) A circular patch with a gradient is perceived as 3D. The surrounding patches are observed as bulges, and the central patch is perceived as flat or concave. (b) When the image is turned upside down, the depth interpretation changes: the concave areas are observed as convex, and the convex areas are observed as concave. (c) A shaded surface. (d) When the surface in c is turned upside down, the depth relief is inverted. This is most easily visible in the center of the image, which has changed from convex to concave. (e) A surface where shading has been replaced with line texture. (f) When the textured image in e is turned upside down, the depth relief changes.

Such assumptions about the light source are also made when viewing more complex grayscale images such as in Figure 1(c) and (d). Turning Figure 1(c) upside down, as shown in Figure 1(d), results in a perception where the convex and concave parts of the image change. However, Reichel and Todd (1990) showed that shading is not necessary for the inversion effect. For example, when Figure 1(e) is turned upside down, as shown in Figure 1(f), the image relief changes. The change occurs because the visual system makes the assumption that the image is being viewed from overhead, which means that we perceive images as being backwardly slanted away from us. Because of this, we assume that depth increases with height in the visual field (Reichel and Todd, 1990). Further assumptions were demonstrated by Langer and Bülthoff (2001), whose experiments showed that the visual system assumes global convexity in images. This effect was later confirmed with photorealistic images by Liu and Todd (2004).

Related to the previous depth inversion phenomena, in this study, we observed an image for which inversion causes a change not only in the relief but also in the perceived material properties of the image. Figure 2(a) shows a grayscale photograph of waves. When the image is turned upside down, in Figure 2(b), the 3D properties of the image change. In other words, the convex parts of the image become concave, and the concave parts become convex. Interestingly, the material properties of the surface also change, as the upside-down waves now appear to be completely different. Observations within our research group suggested that some people perceived the inverted image as a rocky surface, whereas others observed it as a microscopic image of human tissue. This effect can also be produced by turning one’s head upside down, as in other similar demonstrations. To explore the differences between interpretations of upright and inverted images, we conducted an experiment in which observers were asked to describe both images.

**Figure 2.**
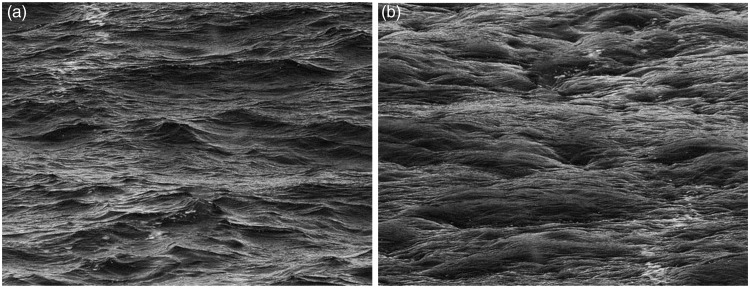
(a) A photograph of waves. (b) Turning the photograph upside down changes both the 3D structure of the scene and the interpretation of its material properties. The upside-down version of the image is most commonly described as a “rocky surface” or a “microscopic image of human tissue.”

The experiment was conducted with 67 observers (51 females and 16 males) who were students at the Institute of Behavioural Sciences, University of Helsinki. The stimuli were displayed on an Eizo ColorEdge CG241W display with a resolution of 1920 pixels in width and 1200 pixels in height. A separate, smaller display was on the table, and it was used for showing the text box used for answering. The viewing distance was 80 cm, and the size of the images was 9.6° × 7.4° of visual angle. Observers viewed the two images in a randomized order and answered the question “What does this image depict?” by writing a free answer with a computer keyboard. The presentation time was not limited.

The results show that inverting the image changed the viewers’ interpretation of the surface material. The leftmost blue bar in Figure 3 shows that all participants recognized the upright image as water. In contrast, there was more variation in the descriptions of the inverted image, as shown by the rightmost bars in Figure 3. Most of the participants (frequency (*f*) = 37 of 67) described the image as a solid material such as rock (*f* = 28) or as a microscopic image of human tissue (*f* = 8). Several participants described the surface as water (*f* = 26), but most of these (*f* = 19 of 26) described it as rapids, not as waves. There were also four participants who said that the surface could be either water or a solid surface. These four answers were omitted from further analysis.

**Figure 3.**
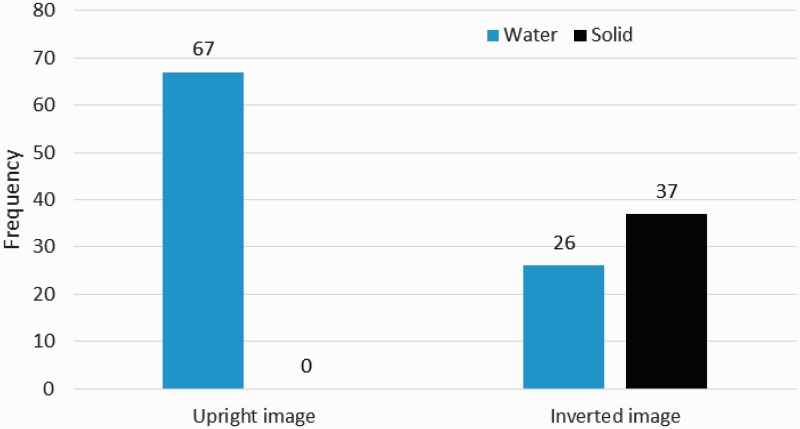
Frequency of answers belonging to the water or solid categories. Blue bars indicate the frequency of water category answers, and black bars indicate the frequency of solid category answers. The leftmost bars show the results for the upright image, and the rightmost bars show the results for the inverted image. The numbers above the bars indicate the frequency of answers. Note that in the inverted image condition, four answers have been removed because the participants indicated both categories.

Most likely, a number of variables affected the result. First, in one-half of the experimental cases, the participants observed the waves image first. This initial exposure might have primed them to interpret the inverted image as water. Second, the foam at the top left part of Figure 2(a) is a cue for water that stays invariant in the inverted image (bottom left area of Figure 2(b)). Using a stimulus without this additional cue might reduce the number of people who interpret the inverted image as water. Third, the method of giving free answers increased the variability in the results. The effect needs to be quantified with a more exact methodology.

This phenomenon, which we call the inverted waves effect, is probably related to the visual characteristics of fluids. Paulun, Kawabe, Nishida, & Fleming (2015) noticed that the perceived viscosity of fluids varies systematically with the 2D shape statistics of images. Their experimental stimuli consisted of computer simulations of fluid pouring from a source into a reservoir, so the stimulus properties were different from our wave images, which are interpreted as having specific visual structures (Kung & Richards, 1988). It would be interesting to further investigate what these visual properties are and how they are used for fluid categorization in images with waves. We noted that the effect is difficult to reproduce with wave images found from Internet searches. An example of this is shown in Figure 4(a), which does not change its material properties when turned upside down, as shown in Figure 4(b). This suggests that there are specific conditions for the effect, and these need to be defined in further research.

**Figure 4.**
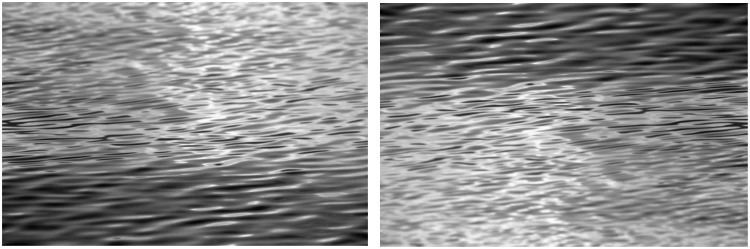
(a) A photograph of waves. (b) Turning this photograph upside down does not change the 3D interpretation or the interpretation of the surface material. (Images are in the public domain.)
